# Antenatal diagnosis of absence of septum pellucidum

**DOI:** 10.1002/ccr3.2666

**Published:** 2020-02-05

**Authors:** Imane Ben M'Barek, Mikael Tassin, Agnes Guët, Isabelle Simon, Valerie Mairovitz, Laurent Mandelbrot, Olivier Picone

**Affiliations:** ^1^ Service de Gynécologie‐Obstétrique Assistance Publique‐Hôpitaux de Paris Hôpital Louis Mourier Colombes France; ^2^ Université de Paris Paris France; ^3^ Service de Néonatalogie and Service de Pédiatrie Assistance Publique‐Hôpitaux de Paris Hôpital Louis Mourier Colombes France; ^4^ Service d’Imagerie Assistance Publique‐Hôpitaux de Paris Hôpital Louis Mourier Colombes France

**Keywords:** cerebral MRI, limbic system, prenatal diagnosis, Septal agenesis, septo‐optic dysplasia, Septum pellucidum

## Abstract

The absence of septum pellucidum (ASP) is a rare disease, which affects the structure of the brain. It is either isolated or associated with various congenital brain malformations. The diagnosis of ASP can be performed by second‐trimester ultrasound. When the ASP is isolated, prenatal counseling is optimistic regarding neurological outcome, but there is a 20% risk of septo‐optic dysplasia in the neonate.

## Graphical abstract

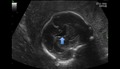

## 1 INTRODUCTION

The septum pellucidum (SP) is formed during the 8th week of embryonic development in the folds of primordium hippocampus and is crossed by corpus callosum (CC). It is made up of two membranes which represent the lateral ventricle's wall: the laminae.^1^ It is part of the limbic system and is filled in with cerebrospinal fluid.

The absence of SP (ASP) is a rare cerebral disease whose prevalence in a general population is between 2 and 3/100 000 according to several studies.^2^, ^3^, ^4^ ASP is either a septal disruption or the result of an agenesis of the SP. Also, it can be partial or complete, and congenital or acquired.^5^

The absence of septum pellucidum can be associated with other cerebral anomalies such as CC agenesis, septo‐optic dysplasia, Arnold‐Chiari malformation, schizencephaly, or holoprosencephaly.^3^ In such cases, the prognosis depends mainly on the associated brain anomalies.

On the contrary, the neurological prognosis is poorly known when the ASP is isolated. Thus, prenatal counseling is difficult, in particular regarding the question of whether a termination of pregnancy for fetal anomalies (TOPFA) should be considered. This study aims to close this gap through 4 case reports for which an isolated ASP was diagnosed in the prenatal period.

## 2 CASES REPORTS

The sonographic examinations described below were performed using either an Aloka or Voluson E8 ultrasound machine equipped with 3.5 to 14 MHz abdominal and transvaginal probes. The cases are summarized in Table 1.

**Table 1:** Summary of the cohort of 4 cases of absent septum pellucidum

Case	Gestational age at diagnosis (WG)	Cerebral abnormalities associated detected at US examination	Other morphologic abnormalities detected at US examination	Karyotype, CGH array	MRI at 32 weeks	Gestational age at delivery (WG)	Neurological evaluation at birth	Endocrine evaluation	Final diagnosis
**1**	22	No	No	Not performed	Hypointense pituitary gland	37 + 4	Dysexecutive syndrome	GH and TSH deficiency	Septo‐optic dysplasia
**2**	22 + 5	No	Esophageal atresia, SUA	Normal	Normal	40 + 2	Normal	Normal	ASP and esophageal atresia
**3**	32	No	No	Normal	Normal	39	Normal	Normal	Isolated ASP
**4**	27 + 5	Thin CC	No	Normal	CC dysgenesis, perisylvian microgyria	38	Left monoparesis, language delay	Normal	ASP and opercular dysplasia

Abbreviation: ASP, The absence of septum pellucidum.

### 2.1 Case 1

The mother was a 21‐year‐old primigravida with no medical history but who smoked tobacco during the pregnancy. First‐trimester ultrasound (US) screening was not performed, and second‐trimester screening US was performed late, at 26 weeks of gestation (WG), after which she was referred for a suspicion of ASP.

**Figure 1:**
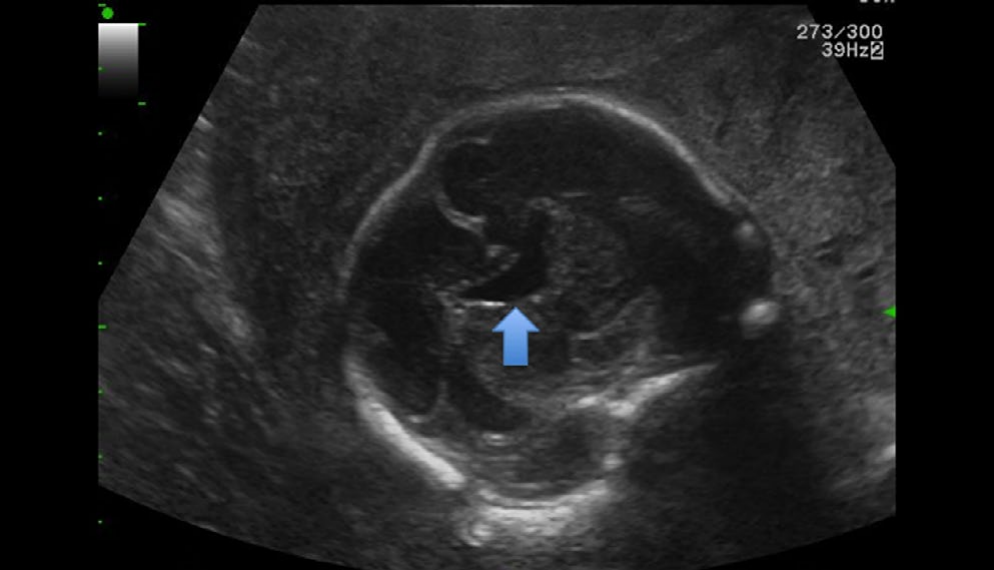
Coronal US performed at 26 WG and 6 d showing the square fused frontal horns

Sonographic examination was performed in our institution at 26 WG and 6 days, and an isolated ASP was diagnosed: The frontal horns of lateral ventricle were fused in the anterior complex (Figure 1). The CC was normal, and there was no ventriculomegaly. The cerebral gyration seemed normal. MRI at 32 WG confirmed the US examination and showed a normal optic track and chiasma. There was a hypointense pituitary gland. The couple did not consent to an amniocentesis.

A full‐term 2960‐g female was born by C‐section, at the mother's request because of breech presentation. The neonate was hypoglycemic despite a normal endocrine dosage. MRI was performed at 2 months due to an abnormal visual behavior. It showed septo‐optic dysplasia and suspected frontal schizencephalic clefts. A second postnatal MRI was performed because of a head trauma, which showed hypoplasia of the optic track and pituitary gland, and ventricular septum agenesis. There was also a suprasellar cyst that shifted the anterior portion of the pituitary stalk and contained an ectopic posterior pituitary gland. Biological testing showed hyponatremia, as well as thyroid‐stimulating hormone (TSH) and growth hormone deficiencies. Clinically, the child had visual dyspraxia. The diagnosis of septo‐optic dysplasia was confirmed, and a multidisciplinary follow‐up was organized. The child is now 5 years old, has a dysexecutive syndrome, and attends a school for children with disabilities.

### 2.2 Case 2

The patient was a healthy 19‐year‐old primigravid woman with no medical history, married to her first cousin. She was referred at 22 WG because of a suspicion of isolated ASP at the second‐trimester screening US.

**Figure 2:**
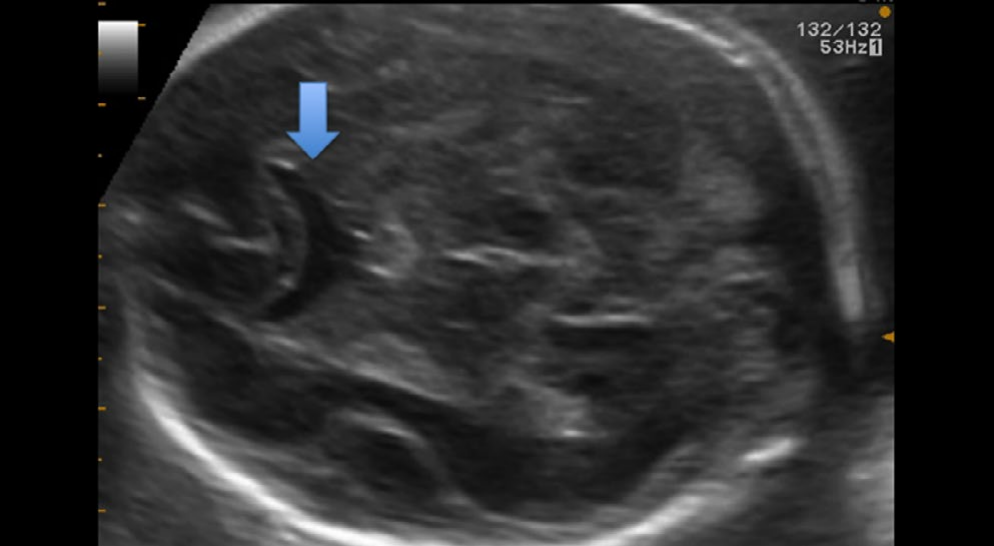
Axial section at 22 WG and 5 d. Anterior horns are merged

The first‐trimester US at 11 WG and 6 days was normal, with a nuchal translucency (NT) of 1.5 mm for a craniocaudal length (CCL) of 50.3 mm. A 2D US examination was performed at 22 WG and 5 days (Figure 2) and revealed an ASP. The associated cerebral imaging was normal, but there was a single umbilical artery, a right aortic arch, a small stomach, and a polyhydramnios. Esophageal atresia was suspected. An amniocentesis was performed, and karyotype and CGH array were normal. MRI performed at 32 WG confirmed that there was an isolated ASP. The cerebral gyrations as well as the CC, the chiasma, the optic track, and the MRI signal for the pituitary gland were normal for the gestational age. MRI was also suggestive of esophageal atresia.

A full‐term 2830‐g male was born after normal delivery. Grade 3 esophageal atresia was confirmed, and surgery was performed on the first day postnatally. From a neurological standpoint, an MRI at 12 months revealed thoracic and lumbar syringomyelia as well as an ASP. The neurological assessment was normal. The child is now 4 years old and has normal development.

### 2.3 Case 3

The patient was a 33‐year‐old primigravida, who was obese and had uterine fibromas, with no other medical history. She was referred at 32 WG because of a suspicion of ASP.

**Figure 3:**
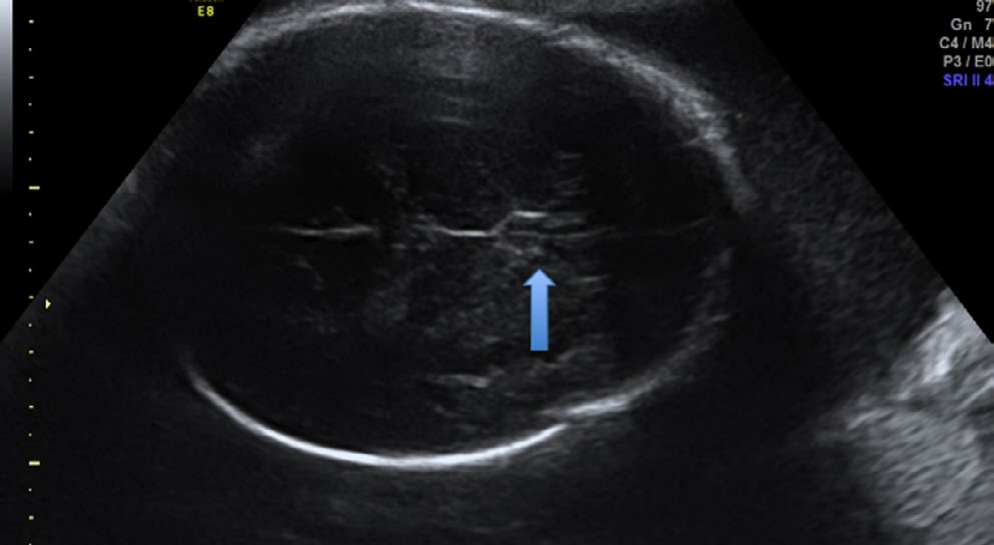
Axial US at 32 WG and 4 d. The wall of the ventricle can be mistaken for the columns of the fornix

**Figure 4:**
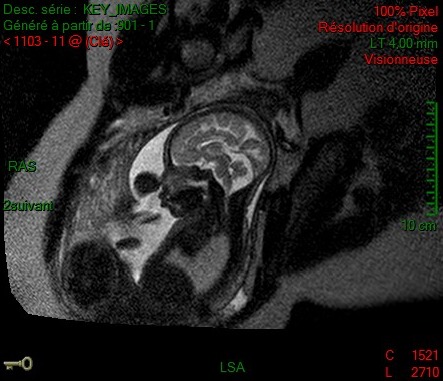
Sagittal MRI performed at 32 WG. Short corpus callosum

**Figure 5:**
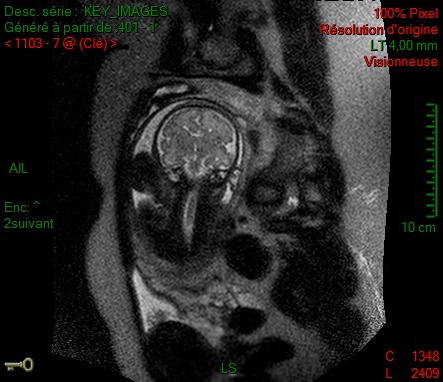
T2‐weighted MRI at 32 WG, coronal view showing perisylvian microgyria on the right side

First‐trimester screening US was normal at 12 WG and 4 days, with a NT of 1.2 mm for a CCL of 63.6 mm, and the second‐trimester screening US was reported as normal. Third‐trimester screening US examination showed a partial isolated ASP (Figures 3,4). The karyotype and CGH array obtained by amniocentesis were normal, and the MRI performed at 32 WG confirmed the isolated ASP (Figure 5). The rest of the examination was normal.

A full‐term 2740‐g was delivered by C‐section because of an obstructing myoma. The endocrine and neurological evaluation performed after birth were both normal. Neurological assessment was normal at 6 months. The child is now 2 years old and has normal neurological development.

### 2.4 Case 4

The patient was a 33‐year‐old healthy multigravida (third pregnancy) with no medical history. She was referred at 27 WG for suspicion of ASP. The couple was not related.

On the first‐trimester US, the NT was 1.4 mm for a CCL of 52 mm. Because of adverse conditions (fibroids) during second‐trimester US, a supplementary scan was performed at 27 WG and 5 days, which diagnosed ASP. It also revealed a thin CC (32.4 mm, which is under the fifth percentile according to Achiron curve.^6^ There was no ventriculomegaly. Fetal karyotype and CGH array obtained by amniocentesis were normal. The MRI performed at 32 WG revealed a midline cerebral abnormality (ASP, CC dysgenesis [Figure 4]) and a cerebral gyration anomaly, with perisylvian polymicrogyria on the right side (Figure 5). An ischemic stroke was suspected. Platelet‐specific antibodies were not found. Despite counseling suggesting a high likelihood of poor neurological outcome, the couple did not request a termination of pregnancy.

A full‐term girl was delivered normally. The neurological assessment was normal after birth. The child is now 2 years old and has a left monoparesis and a language delay. An ASP with bilateral opercular dysplasia has been diagnosed.

## 3 DISCUSSION

In the 4 cases described, an ASP was diagnosed by prenatal US examination. The ASP appeared isolated in terms of prenatal neuro‐imaging in 3 cases; however, one of these was found to have a SOD during the neonatal period.

The septal area is visualized from 18‐20 WG. On an axial section, the SP is an anechoic square with thin walls in the median line of the anterior complex. On a slightly lower section, this anechoic square is crossed in its length by thin echogenic lines, which are the columns of the fornix.^1^, ^7^, ^8^ They can be mistaken for the cavum pellucidum: This can create false negatives (Figure 3).^1^, ^9^ Complementary coronal and axial sections are needed if in doubt.^8^ In case of ASP, the anterior horns of lateral ventricles are fused on the median line and look like a “butterfly wing.” Also, cases of ASP often have ventriculomegaly and a horizontalization of the ventricle's roof. In some cases, the CC is thin and the columns of the fornix are not merged.^8^

Academic societies recommend visualizing the anterior complex during the morphological US examination,^10^ which is usually performed around 22 WG in France. There are few reports on ASP diagnosed in utero.^11^, ^12^, ^13^, ^14^ Their diagnoses are generally performed during the third‐trimester US.^11^, ^12^, ^14^ In two of our cases and in Pilliod's series,^13^ diagnoses were made earlier during the morphological US. In the two other cases, the diagnosis was delayed because of adverse conditions (fibromas and maternal obesity). Indeed, the evaluation of fetal anatomy is more difficult on overweight women because of the impaired acoustic window,^15^, ^16^, ^17^, ^18^ and the detection of anomalous fetuses is 20% lower compared to patients with normal BMI.^16^ The US velocity setting can be lowered to less than 1540 m/s 15 to improve the image quality of mid‐trimester fetal sonography in obese women. In France, a recent investigation in perinatal health showed that more than 30% of pregnant women are overweight or obese.^19^

When an ASP is diagnosed, the prognostic evaluation of the neurological outcome depends on the associated cerebral anomalies. Thus, the challenge for prenatal diagnosis is to determine whether the ASP is isolated or not. Thus, the fetal investigation should include an amniocentesis for karyotyping and CGH array and a fetal cerebral MRI, which is most informative around 32 WG. It is worth noting that the prenatal and postnatal imaging are very similar.^13^ In our fourth case, fetal MRI showed that the ASP was due to a septal disruption which is the result of a cerebral stroke: the ASP was only the tip of the iceberg.

The neuropsychological and the neurological evaluations are generally good in the case of an isolated ASP.^10^, ^11^, ^13^ Damaj et al reported the largest cohort of patient with isolated ASP diagnosed during the prenatal period, with follow‐up for neurological and cognitive‐behavioral outcomes. Among 17 patients, 3 had behavioral and neurological problems, which were visual‐spatial dyspraxia and language delay. When an isolated ASP is suspected, prenatal counseling is difficult because SOD cannot be dismissed in utero, since it is not detected by prenatal cerebral imaging by US and MRI in one‐half of cases.^3^ SOD was determined after birth 1 of 4 cases in our cohort, in 3 of 17 cases in the series of Damaj et al^11^ and 2 of 8 cases in the series of Pilliod et al^13^ The spectrum of SOD is characterized by hypoplasia of the chiasma and optical tracks, hypopituitarism and midline cerebral anomalies. There is an ASP in 60% of the cases. The symptoms of the pathology are variable, and its prognosis depends on their severity.^12^, ^20^, ^21^ The chiasma, optic track, and pituitary gland are hard to visualize by US because of their thin structure.^22^ Hence, a cerebral MRI should be performed during the neonatal period when an isolated ASP is suspected. Lepinard et al^21^ proposed to measure estriol concentrations in maternal blood and urine to determine if the pituitary gland is damaged. Indeed, the resulting adrenal insufficiency causes a decrease of estriol concentrations in the mother's blood in case of fetal ACTH deficiency. Those tests are not routinely performed, and the severity of SOD is not correlated with the maternal estriol concentration.

Isolated ASP has no known genetic origin. In contrast, the etiology of SOD, while it is still poorly known, has genetic cause involving genes such as HESX1. HESX1 is a homobox gene that controls pituitary organogenesis and in particular the formation of the pituitary precursor, the Rathke's pouch.^20^ Thus, an amniocentesis should be performed when there are morphological anomalies on the sonographic examination.

Given its role in the limbic system, the septum pellucidum (SP) plays a role in the attention and the emotions. At 6 months of age, 85% of children no longer have a SP.^9^ Many studies have linked psychotic disorders such as schizophrenia with ASP.^5^, ^23^, ^24^ The meta‐analysis by Trzesniak et al^25^ confirms this link when the septum is large.

In conclusion, ASP is a rare anomaly that can be diagnosed at second‐trimester US examination, when the anterior complex is carefully analyzed. In case of poor technical conditions (overweight, fibroids, fetal presentation, anamnios, …), an intermediate US examination at 26 WG should be performed to complete the screening. Prenatal investigations in case of ASP should include an amniocentesis for karyotyping and CGH array, and a fetal cerebral MRI in order to determine whether the ASP is isolated or not. Antenatal counseling may be optimistic when the ASP is isolated, but must mention the 20% risk of a diagnosis of SOD in the neonatal period.

## CONFLICT OF INTEREST

None declared.

## AUTHOR CONTRIBUTIONS

Dr I. Ben M’Barek: drafted the initial manuscript. Dr M. Tassin, Dr A.Guët, Dr I.Simon, Dr V. Mairovitz, Pr L. Mandelbrot and Pr O. Picone: were involved in the management of patients, in revising critically the manuscript for important intellectual content. All authors approved the final manuscript as submitted.

## References

[1] Callen PW , Callen AL , Glenn OA , Toi A . Columns of the fornix, not to be mistaken for the cavum septi pellucidi on prenatal sonography. J Ultrasound Med. 2008;27(1):25‐31. 18096727 10.7863/jum.2008.27.1.25

[2] Barkovich AJ , Norman D . Absence of the septum pellucidum: a useful sign in the diagnosis of congenital brain malformations. AJR Am J Roentgenol. 1989;152(2):353‐360. 2783514 10.2214/ajr.152.2.353

[3] Malinger G , Lev D , Kidron D , Heredia F , Hershkovitz R , Lerman‐Sagie T . Differential diagnosis in fetuses with absent septum pellucidum. Ultrasound Obstet Gynecol. 2005;25(1):42‐49. 15593321 10.1002/uog.1787

[4] Bruyn GW . Agenesis septi pellucidi, cavum septi pellucidi, cavum Vergae and cavum veli interpositi In: Vinken PJ, Bruyn GW, eds. Congenital Malformations of the Brain and Skull Part 1. Amsterdam, North Holland; 1977: 299‐336.

[5] Schaefer GB , Bodensteiner JB , Thompson JN . Subtle anomalies of the septum pellucidum and neurodevelopmental deficits. Dev Med Child Neurol. 1994;36(6):554‐559. 7516298 10.1111/j.1469-8749.1994.tb11888.x

[6] Achiron R , Achiron A . Development of the human fetal corpus callosum: a high‐resolution, cross‐sectional sonographic study. Ultrasound Obstet Gynecol. 2001;18(4):343‐347. 11778993 10.1046/j.0960-7692.2001.00512.x

[7] Winter TC , Kennedy AM , Byrne J , Woodward PJ . The cavum septi pellucidi. J Ultrasound Med. 2010;29(3):427‐444. 20194938 10.7863/jum.2010.29.3.427

[8] Garel C , Moutard M‐L . Main congenital cerebral anomalies: how prenatal imaging aids counseling. Fetal Diagn Ther. 2014;35(4):229‐239. 24577226 10.1159/000358519

[9] Pilu G , Tani G , Carletti A , Malaigia S , Ghi T , Rizzo N . Difficult early sonographic diagnosis of absence of the fetal septum pellucidum. Ultrasound Obstet Gynecol. 2005;25(1):70‐72. 15619322 10.1002/uog.1786

[10] Cagneaux M , Guibaud L . From cavum septi pellucidi to anterior complex: how to improve detection of midline cerebral abnormalities. Ultrasound Obstet Gynecol. 2013;42(4):485‐486. 23674330 10.1002/uog.12505

[11] Damaj L , Bruneau B , Ferry M , et al. Pediatric outcome of children with the prenatal diagnosis of isolated septal agenesis. Prenat Diagn. 2010;30(12–13):1143‐1150. 20936603 10.1002/pd.2628

[12] Malinger G , Lev D , Oren M , Lerman‐Sagie T . Non‐visualization of the cavum septi pellucidi is not synonymous with agenesis of the corpus callosum. Ultrasound Obstet Gynecol. 2012;40(2):165‐170. 22689012 10.1002/uog.11206

[13] Pilliod RA , Pettersson DR , Gibson T , et al. Diagnostic accuracy and clinical outcomes associated with prenatal diagnosis of fetal absent cavum septi pellucidi. Prenat Diagn. 2018;38(6):395‐401. 29532939 10.1002/pd.5247

[14] García‐Arreza A , García‐Díaz L , Fajardo M , Carreto P , Antiñolo G . Isolated absence of septum pellucidum: prenatal diagnosis and outcome. Fetal Diagn Ther. 2013;33(2):130‐132. 22572040 10.1159/000338009

[15] Chauveau B , Auclair C , Legrand A , et al. Improving image quality of mid‐trimester fetal sonography in obese women: role of ultrasound propagation velocity. Ultrasound Obstet Gynecol. 2018;52(6):769‐775. 29363850 10.1002/uog.19015

[16] Dashe JS , McIntire DD , Twickler DM . Effect of maternal obesity on the ultrasound detection of anomalous fetuses. Obstet Gynecol. 2009;113(5):1001‐1007. 19384114 10.1097/AOG.0b013e3181a1d2f5

[17] Fuchs F , Houllier M , Voulgaropoulos A , et al. Factors affecting feasibility and quality of second‐trimester ultrasound scans in obese pregnant women. Ultrasound Obstet Gynecol. 2013;41(1):40‐46. 23023941 10.1002/uog.12311

[18] Thornburg LL , Miles K , Ho M , Pressman EK . Fetal anatomic evaluation in the overweight and obese gravida. Ultrasound Obstet Gynecol. 2009;33(6):670‐675. 19479682 10.1002/uog.6401

[19] Blondel B , Coulm B , Bonnet C , Goffinet F , Le Ray C . Trends in perinatal health in metropolitan France from 1995 to 2016: results from the French National Perinatal Surveys. J Gynecol Obstet Hum Reprod. 2017;46(10):701‐713. 29031048 10.1016/j.jogoh.2017.09.002

[20] McCabe MJ , Alatzoglou KS , Dattani MT . Septo‐optic dysplasia and other midline defects: the role of transcription factors: HESX1 and beyond. Best Pract Res Clin Endocrinol Metab. 2011;25(1):115‐124. 21396578 10.1016/j.beem.2010.06.008

[21] Lepinard C , Coutant R , Boussion F , et al. Prenatal diagnosis of absence of the septum pellucidum associated with septo‐optic dysplasia. Ultrasound Obstet Gynecol. 2005;25(1):73‐75. 15593257 10.1002/uog.1807

[22] Blondiaux E , Garel C . Fetal cerebral imaging – ultrasound vs. MRI: an update. Acta Radiol. 2013;54(9):1046‐1054. 23012483 10.1258/ar.2012.120428

[23] Morgan SA . Absence of the septum pellucidum: overlapping clinical syndromes. Arch Neurol. 1985;42(8):769. 4026611 10.1001/archneur.1985.04210090033010

[24] Bodensteiner JB , Schaefer GB , Craft JM . Cavum septi pellucidi and cavum vergae in normal and developmentally delayed populations. J Child Neurol. 1998;13(3):120‐121. 9535237 10.1177/088307389801300305

[25] Trzesniak C , Oliveira IR , Kempton MJ , et al. Are cavum septum pellucidum abnormalities more common in schizophrenia spectrum disorders? A systematic review and meta‐analysis. Schizophr Res. 2011;125(1):1‐12. 20965698 10.1016/j.schres.2010.09.016

